# A Comprehensive Evaluation of the Performance of Prediction Algorithms on Clinically Relevant Missense Variants

**DOI:** 10.3390/ijms23147946

**Published:** 2022-07-19

**Authors:** Erda Qorri, Bertalan Takács, Alexandra Gráf, Márton Zsolt Enyedi, Lajos Pintér, Ernő Kiss, Lajos Haracska

**Affiliations:** 1 HCEMM-BRC Mutagenesis and Carcinogenesis Research Group, Institute of Genetics, Biological Research Centre, H-6726 Szeged, Hungary; qorri.erda@brc.hu (E.Q.); takacs.bertalan@brc.hu (B.T.); graf.alexandra@brc.hu (A.G.); ekiss@brc.hu (E.K.); 2 Faculty of Science and Informatics, Doctoral School of Biology, University of Szeged, P.O. Box 427, H-6720 Szeged, Hungary; 3 Delta Bio 2000 Ltd., H-6726 Szeged, Hungary; marton.enyedi@deltabio.eu (M.Z.E.); lajos.pinter@deltabio.eu (L.P.)

**Keywords:** benchmark, ClinVar, *BRCA*1, *BRCA*2, type 1 circularity, prediction algorithms

## Abstract

The rapid integration of genomic technologies in clinical diagnostics has resulted in the detection of a multitude of missense variants whose clinical significance is often unknown. As a result, a plethora of computational tools have been developed to facilitate variant interpretation. However, choosing an appropriate software from such a broad range of tools can be challenging; therefore, systematic benchmarking with high-quality, independent datasets is critical. Using three independent benchmarking datasets compiled from the ClinVar database, we evaluated the performance of ten widely used prediction algorithms with missense variants from 21 clinically relevant genes, including *BRCA*1 and *BRCA*2. A fourth dataset consisting of 1053 missense variants was also used to investigate the impact of type 1 circularity on their performance. The performance of the prediction algorithms varied widely across datasets. Based on Matthews Correlation Coefficient and Area Under the Curve, SNPs&GO and PMut consistently displayed an overall above-average performance across the datasets. Most of the tools demonstrated greater sensitivity and negative predictive values at the expense of lower specificity and positive predictive values. We also demonstrated that type 1 circularity significantly impacts the performance of these tools and, if not accounted for, may confound the selection of the best performing algorithms.

## 1. Introduction

The use of high-throughput technologies such as next-generation sequencing (NGS) has become routine practice in both cancer research and clinical laboratories in the detection of germline and somatic mutations alike [1]. Its robust performance and extensive application range have made NGS the foremost component of personalized cancer treatment [2]. This accelerated adaptation of NGS in clinical settings has led to the identification of thousands of variants whose effects on protein function, and, ultimately, on patients′ risk of developing cancer, are unknown [3]. The interpretation of variants of uncertain significance (VUS) represents a major challenge for clinicians who, in the absence of relevant functional and clinical information, are unsure of the potential health implications of these variants [4]. As a result, VUS may often be excluded from medical reports [5]. While significant efforts have been made to develop functional assays to classify these variants, experimental characterization is often tedious and time-consuming. Moreover, given the dramatic rise in the number of the identified variants, it may not be the most viable option, especially in the case of somatic mutations [5].

Over the last two decades, a multitude of computational tools have been developed to address this emerging issue. These prediction algorithms are widely used as evidence to prioritize and select novel variants of unknown significance for in vivo and in vitro functional assays [6]; they are integrated into NGS bioinformatics pipelines [7], and some of them, such as Polyphen2 [8], SIFT [9], and MutationTaster [10], are integrated into commercially available interfaces and are routinely used in clinical diagnostics [11].

To distinguish between deleterious and neutral variants, these tools employ a variety of features, including sequence homology; evolutionary conservation; physicochemical differences between wild-type and mutant amino acids; structural information; protein interactomes; or a combination of the abovementioned features [8,9,12,13,14,15]. In general, prediction algorithms can be divided into three major categories: (i) evolutionary conservation-based; (ii) consensus-based; and (iii) machine learning-based methods [16].

Moreover, to standardize the process of variant classification, several guidelines have been published. The guidelines issued by the American College of Medical Genetics and Genomics/Association for Molecular Pathology (ACGM/AMP) recommend the cautious use of multiple prediction algorithms for variant interpretation, advising that computational analysis can only be considered as supporting evidence if the algorithms used are in agreement and provide the same results [17]. Nonetheless, several studies have demonstrated that one major pitfall of this approach is the discordance between algorithms, which entails that the computational evidence must not be considered and, consequently, cannot be utilized as evidence for clinical decision-making [18,19,20]. Furthermore, Ghosh and colleagues reported an additional discordance category, “false concordance”, where prediction algorithms are in agreement with each other but contradict evidence from other sources [19].

In addition, other guidelines, such as those established by the Association for Clinical Genomic Science (ACGS), suggest that meta-predictors will likely replace the use of multiple prediction algorithms [21]. Meta-predictors are a class of variant prediction algorithms that combine the output of several independent prediction methods to discriminate between disease-associated and neutral variants [15]. These tools integrate feature elements from various predictors into machine learning algorithms, such as REVEL [14], META-SNP [15], BayesDel [22], PredictSNP [23], GAVIN [24], and ClinPred [25].

The performance of prediction algorithms is typically evaluated utilizing datasets composed of variants of known clinical significance. These variants are commonly retrieved from public online databases such as ClinVar [26], OncoKB [27], and the Human Mutation Database (HGMD) [28]. Additionally, the publicly available variant database VariBench is a widely used source of curated and high-quality training and benchmarking datasets [29]. Numerous studies have shown that the performance of the prediction algorithms varies depending on the testing dataset used; therefore, they may not perform as well as anticipated when utilized to classify novel variants [20,30]. Another critical caveat is the inherent bias introduced by the utilization of the same variants to train and test the algorithm′s performance, known as type 1 circularity. Type 1 circularity, as described by Grim and colleagues, occurs when there is a substantial overlap between the datasets used to train and benchmark these prediction algorithms, resulting in an overestimation and artificial inflation of their true performance [31]. Although many authors have addressed type 1 circularity [20,32,33,34], the process has often proven to be challenging, since training datasets of the computational methods are not always made publicly available. Additionally, the performance of the computational tools is subject to frequent updates, as developers often modify the algorithms by incorporating additional features or improving existing ones. As a result, systematic, independent, and comparative analyses of the performance of these algorithms, such as the one presented here, are necessary to assist the end-user in selecting the best methods for their needs.

Considering the preponderance of prediction algorithms over the last two decades and the increasing evidence supporting the use of meta-predictors over a concordance-based approach [21], we aimed to evaluate and compare the performance of ten widely used prediction algorithms, including eight individual algorithms (Polyphen-2-HumDiv [8], Polyphen-2-HumVar [8], SIFT [9], PMut [12], PROVEAN [13], PhD-SNP [35], SNPs&GO [36], and PANTHER-PSEP [37]) and two meta-predictors (META-SNP [15] and PredictSNP [23]). To carry out this assessment, we compiled three high-quality benchmarking datasets from the ClinVar database [26]. On the main dataset, consisting of 404 missense variants, our results showed that the performance of the prediction algorithms varied considerably. Additionally, prompted by our initial results and the high number of VUS reported for the *BRCA*1 and *BRCA*2 genes, we further compared the performance of the selected prediction algorithms in predicting missense variants derived from these two genes. Lastly, we also explored the impact of type 1 circularity on the performance of seven machine learning-based prediction algorithms, namely, HumDiv (Polyphen-2), HumVar (PolyPhen-2), PhD-SNP, SNPs&GO, META-SNP, PredictSNP, and PMut. For this task, we compiled a benchmarking dataset that shared different degrees of overlap with the training dataset(s) of the abovementioned predictors. As expected, the performance of the prediction algorithms across all of the calculated metrics increased remarkably.

## 2. Results

### 2.1. Benchmarking Datasets and Evaluation on the Expert Panel Dataset

To evaluate and compare the performance of the prediction algorithms PROVEAN, META-SNP, SIFT, Polyphen-2-HumDiv (PP-2-HumDiv), Polyphen-2-HumVar (PP-2-HumVar), SNPs&GO, PredictSNP, PhD-SNP, PANTHER-PSEP, and PMut, we generated three independent benchmarking datasets: (i) the expert panel dataset (EP) (Supplementary Table S1) containing 404 missense variants from 21 clinically relevant genes, such as *BRCA*1, *BRCA*2, *MSH*2, *MSH*6, *MLH*1, *MYH*7, etc. (for a complete list of the genes, refer to Supplementary Table S2); (ii) the BRCA1 dataset composed of 151 missense variants from the *BRCA*1 gene (Supplementary Table S3); and (iii) the BRCA2 dataset composed of 134 missense variants from the *BRCA*2 gene (Supplementary Table S4).

Compiling a proper benchmarking dataset is essential to conduct an unbiased and accurate evaluation of the performance of the prediction algorithms. To this end, in order to ensure that the compiled datasets were comprised of variants of high confidence, only those ClinVar variants were considered that had been reviewed by an expert panel and/or reported by multiple submitters, with concordant evidence regarding their clinical significance.

Moreover, although none of the prediction algorithms were directly trained on ClinVar data, the possibility of overlapping variants existing between their training datasets and our benchmarking datasets could not be completely ruled out. Therefore, we retrieved and meticulously examined the training datasets of the algorithms and removed all variants that overlapped with our benchmarking datasets.

Initially, the performance of the prediction algorithms was evaluated on the EP dataset composed of 199 pathogenic and 205 benign missense variants from 21 clinically relevant genes. Of the 404 variants, PANTHER-PSEP and PMut did not return prediction scores for 38 (9.4%) and 12 (2.97%) variants, respectively. PANTHER-PSEP was unable to generate prediction scores for the following genes: *MTOR* (P42345), *MYH*7 (P12883), and *SLC26A*4 (O43511), possibly due to the fact that the input sequence did not match any of the sequences for which a gene family is available in the PANTHER database. On the other hand, PMut did not generate prediction scores for the *USH2A* gene (O75445). A possible explanation could be the fact that this gene was not present in the online repository of PMut. Therefore, to ensure a fair comparison of the prediction algorithms and in an effort to avoid potential bias in the interpretation of the results, only tools with ≤3% missing values were included in the downstream analysis. Accordingly, PANTHER-PSEP, whose missing values exceeded 9%, was excluded from further analysis in this dataset.

**Figure 1:**
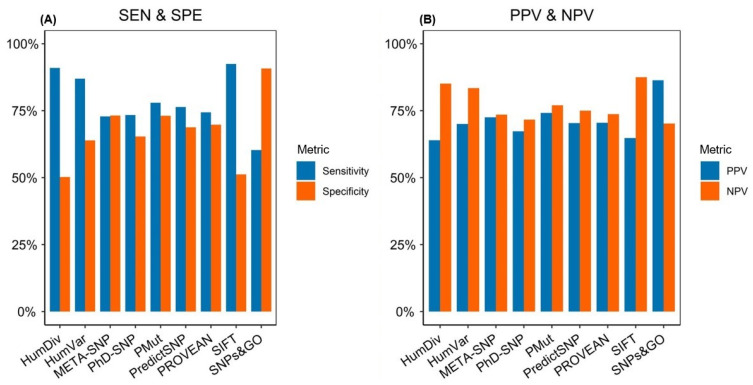
Comparison of the performance metrics of the prediction algorithms on the EP dataset. The EP dataset was composed of 404 expert panel-reviewed missense variants from 21 clinically relevant genes. Variants were retrieved from the ClinVar datasets. (**A**) SEN and SPE; (**B**) PPV and NPV. SEN, sensitivity; SPE, specificity; PPV, positive predictive value; NPV, negative predictive value. PANTHER-PSEP was omitted from further analysis in this dataset.

When analyzed using pathogenicity thresholds as recommended in the literature, we found that none of the prediction algorithms could achieve 100% sensitivity and/or specificity (Supplementary Table S5). The sensitivity values varied considerably among the prediction algorithms, ranging from 60.30% to 92.46% with a median value of 76.38% (Figure 1A). Two predictors, SIFT and PP-2-HumDiv, displayed sensitivities >90%, whereas SNPs&GO showed the lowest sensitivity (60.30%). Comparatively, the specificity values, ranging from 50.24% to 90.73% and with a median value of 68.78%, were markedly lower than the sensitivity values (Figure 1A). SNPs&GO showed the highest specificity (90.73%), while PP-2-HumDiv (50.24%) and SIFT (51.22%) showed the lowest.

In the next step, we calculated the PPV and NPV values for each prediction algorithm. In this analysis, we found that the NPV values, ranging from 70.19% to 87.50% (median of 75.00%), were overall higher than the PPV values, ranging from 63.96% to 86.33% (median of 70.37%). Three predictors—SIFT, PP-2-HumDiv, and PP-2-HumVar—showed NPV values >80%. Interestingly, we observed that SNPs&GO simultaneously achieved the highest PPV (86.33%) and the lowest NPV (70.19%) of all of the prediction algorithms (Figure 1B). SNPs&GO showed excellent performance in accurately classifying benign variants (186/205); however, it also generated the highest number of false negatives among the prediction algorithms (Supplementary Table S6). Furthermore, according to our results, SNPs&GO was the most accurate predictor (ACC = 75.74%), followed by PMut (ACC = 75.51%) and PP-2-HumVar (ACC = 75.25%). PhD-SNP was the least accurate of the algorithms, with an accuracy value of 69.31%. Regarding the meta-predictors, META-SNP (73.02%) showed higher accuracy than PredictSNP (72.52%), ranking 4th.

**Figure 2:**
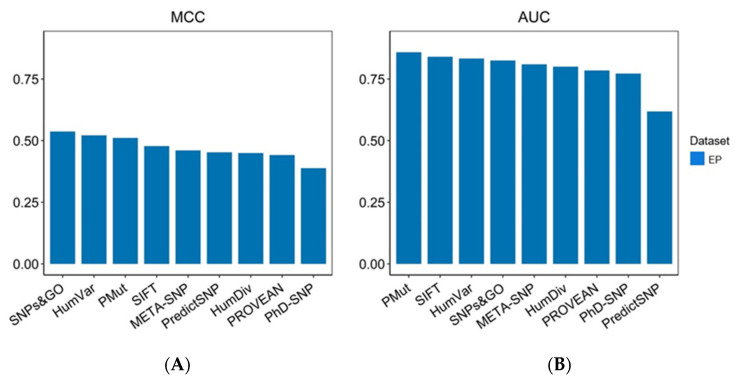
Distribution of the AUC and MCC values of the prediction algorithms on the EP dataset. The EP dataset was composed of 404 missense variants retrieved from the ClinVar database, of which 199 were pathogenic and 205 were benign. (**A**) MCC; (**B**) AUC. MCC, Matthews correlation coefficient; AUC, area under the curve. PANTHER-PSEP was omitted from further analysis on the EP dataset due to the large number of missing values.

Furthermore, based on both the MCC and AUC values, SNPs&GO (MCC = 0.54; AUC = 0.83), PMut (MCC = 0.51; AUC = 0.86), and PP-2-HumVar (MCC = 0.52; AUC = 0.83) outperformed the other prediction algorithms, displaying an above-average performance, whereas PhD-SNP by contrast was the poorest performing prediction algorithm (MCC = 0.39; AUC = 0.77) (Figure 2A,B). Overall, the MCC values ranged from 0.39 to 0.54, with a median of 0.46, whereas the AUC values varied from acceptable (0.62) to excellent (0.86) (Supplementary Figure S1).

Interestingly, the two meta-predictors META-SNP and PredictSNP displayed an overall intermediate performance, ranking fifth and sixth with MCCs of 0.46 and 0.45, respectively. Based on AUCs, META-SNP outperformed PredictSNP, ranking fifth (AUC = 0.81), as compared to PredictSNP, which showed the lowest AUC value among the tools (AUC = 0.62). Moreover, it is pertinent to note that from the two most widely used prediction algorithms in clinical diagnostics, PP-2-HumVar showed an overall better performance than SIFT in this dataset, ranking second in terms of MCCs and third in terms of AUCs (Figure 2A,B). SIFT, on the other hand, ranked fourth in terms of MCCs and second in terms of AUCs. Overall, these predictors displayed high sensitivities (SIFT = 92.46%; PP-2-HumVar = 86.93%) but were offset by poorer specificities (SIFT = 51.22%; PP-2-HumVar = 63.90%) (Figure 1A).

### 2.2. Evaluation on the BRCA1 Dataset

Upon examining the predictions made by the computational tools for each gene in the EP dataset, we observed notable differences in their ability to correctly classify missense variants in the *BRCA*1 and *BRCA*2 genes. Prompted by the fact that mutations occurring in the *BRCA*1 and *BRCA*2 genes account for 5–10% of hereditary breast cancer cases [38] and as much as 80% of the variants identified in these genes remain VUS [39], we further evaluated the performance of the prediction algorithms using two benchmarking datasets composed of BRCA1 and BRCA2 variants.

First, the ten prediction algorithms were evaluated on the BRCA1 dataset consisting of 151 variants, 59 of which were pathogenic and 92 benign (Supplementary Table S3). No missing values were reported; therefore, all ten prediction algorithms, including PANTHER-PSEP, were evaluated in this dataset.

**Figure 3:**
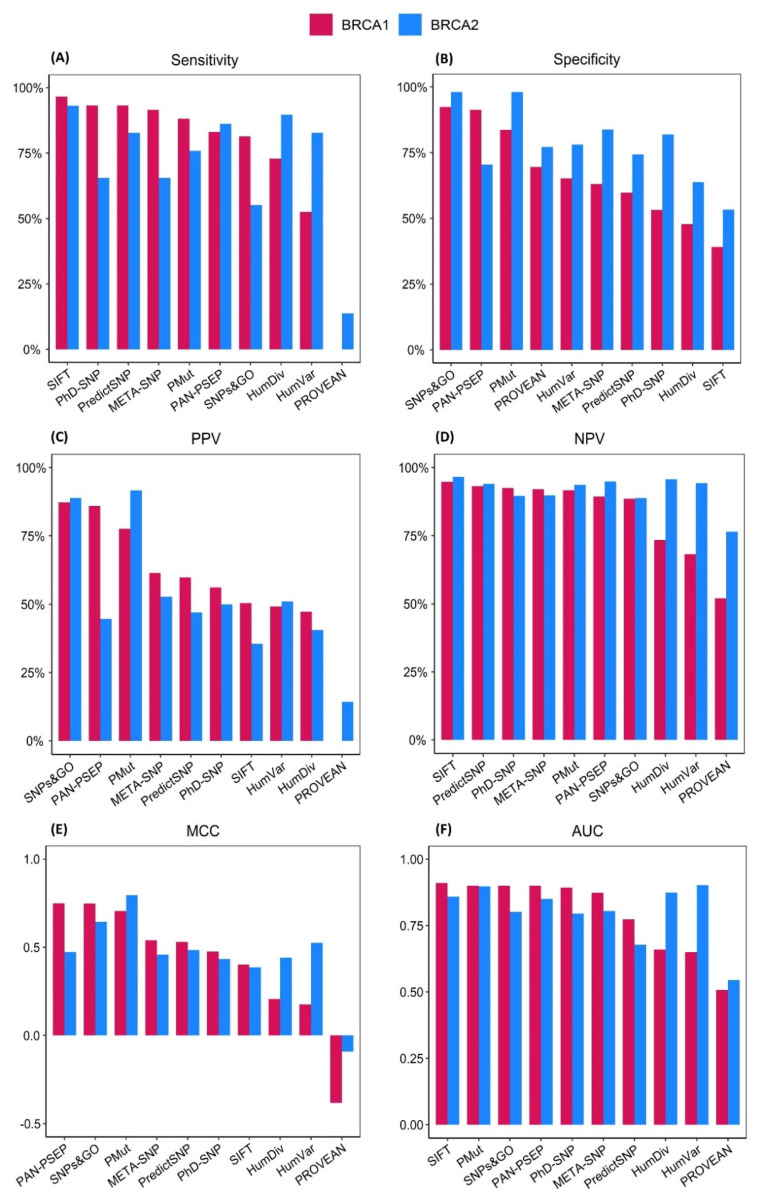
Comparison of the performance metrics of the prediction algorithms in the BRCA1 and BRCA2 datasets. The BRCA1 and BRCA2 datasets consisted of 151 and 134 missense variants from the *BRCA*1 and *BRCA*2 genes, respectively, which were obtained from the ClinVar database. The retrieved variants were assigned either to an expert panel review or to multiple submitters with no conflict of interpretation review status in ClinVar. (**A**) Sensitivity; (**B**) Specificity; (**C**) PPV; (**D**) NPV; (**E**) MCC; (**F**) AUC. The prediction algorithms were arranged from top-performing to poor-performing based on their performance on the BRCA1 dataset. PAN-PSEP, PANTHER-PSEP; PPV, positive predictive value; NPV, negative predictive value; MCC, Matthews correlation coefficient; AUC, Area under curve.

We found that for the majority of the prediction algorithms, the sensitivity (ranging from 0.00% to 96.61%; median of 85.59%) and the NPV values (52.03% to 94.74%; median of 90.51%) were relatively higher than the specificity (39.13% to 92.39%; median of 64.13%) and the PPV (ranging from 0.00% to 87.27%; median of 57.95%) values (Figure 3A,B). Four predictors—SIFT, PhD-SNP, PredictSNP, and Meta-SNP—showed sensitivities >90%, while PP-2-HumVar and PROVEAN showed sensitivities <55% (Table 1). Compared to other methods, SNPS&GO and PANTHER-PSEP displayed the highest specificities (92.39%, and 91.30%, respectively), while PP-2-HumDiv and SIFT displayed the lowest (47.83% and 39.13%, respectively).

Furthermore, we observed that five prediction algorithms (SIFT, PredictSNP, PhD-SNP, META-SNP, and PMut) achieved NPV values >90%, but none of the algorithms could achieve PPV values >90%. Interestingly, PROVEAN ranked last in both metrics, with a PPV of 0.00% and a NPV of 52.03% (Table 1).

To determine the best performing algorithms in this dataset, we calculated their MCC and AUC values. Our results showed that the MCCs and AUCs varied considerably among the prediction algorithms (Supplementary Figure S2). MCCs ranged from poor (−0.38) to relatively high (0.75), with a median of 0.50, whereas the AUCs ranged from no discrimination (0.51) to excellent (0.93) and a median of 0.88 (Figure 3E,F).

Considering these values, the top three performing prediction algorithms for the BRCA1 dataset were PANTHER-PSEP (MCC = 0.75; AUC = 0.89), SNPs&GO (MCC = 0.75; AUC = 0.92), and PMut (MCC = 0.71; AUC = 0.93). By contrast, the poorest performing algorithms were HumDiv (MCC = 0.21; AUC = 0.66), HumVar (MCC = 0.18; AUC = 0.65), and PROVEAN (MCC = −0.38; AUC = 0.51).

Interestingly, we noted that PROVEAN performed poorly across all of the evaluated metrics (sensitivity = 0.00%, specificity = 69.57%, PPV = 0.00%, NPV = 52.03%, accuracy = 42.38%, MCC = −0.38, and AUC = 0.51). This suboptimal performance was attributed to PROVEAN′s inability to correctly classify any of the truly pathogenic BRCA1 variants in this dataset (0/59) (Supplementary Table S6).

### 2.3. Evaluation on the BRCA2 Dataset

The performance of the ten prediction algorithms was also evaluated on the BRCA2 benchmarking dataset containing 29 pathogenic and 105 benign variants (Supplementary Table S4). In this dataset, no missing values were reported by the prediction algorithms; therefore, all ten prediction algorithms were included in the downstream analysis.

Similarly to the BRCA1 dataset, the sensitivity (ranging from 13.79% to 93.10%, median of 79.13%) and the NPV values (ranging from 76.42% to 96.55%, median of 93.81%) were notably higher than the specificity (ranging from 55.33% to 98.10%, median of 77.62%) and the PPV values (ranging from 14.29% to 91.67%, median of 48.53%). SIFT showed both the highest sensitivity (93.10%) and the lowest specificity (53.33%) among all of the evaluated computational tools, whereas SNPs&GO showed the highest specificity along with PMut (98.10%) and the second lowest sensitivity (55.17%) (Table 1). Six prediction algorithms—SIFT, PP-2-HumDiv, PANTHER-PSEP, PP-2-HumVar, PredictSNP, and PMut—displayed NPV values exceeding 90%.

Furthermore, we found that PMut had the highest MCC value (0.79), notably outperforming the other predictors, followed only by SNPs&GO, which displayed a MCC of 0.64 (Figure 3E). Overall, the MCC values ranged from −0.09 to 0.79, with a median value of 0.47. Finally, we evaluated the performance of these prediction algorithms by calculating their AUC values (Supplementary Figure S3), which ranged from 0.54 to 0.90, with a median value of 0.82. Compared to the other algorithms, HumVar and PMut had the highest AUCs (0.90), whereas PROVEAN had the lowest AUC value of 0.54 (Figure 3F).

Surprisingly, we noted that PROVEAN performed poorly in predicting pathogenic variants of BRCA2, similarly to those previously reported in the BRCA1 dataset. Only 4 of the 29 pathogenic variants in this dataset were correctly predicted as pathogenic by PROVEAN (Supplementary Table S6).

### 2.4. Assessing the Effect of Type 1 Circularity on the Performance of the Prediction Algorithms

A persistent challenge encountered in studies of this nature is type 1 circularity, which occurs when the benchmarking datasets include variants that the prediction algorithms were trained on. If not properly addressed, type 1 circularity can artificially enhance the performance of the prediction algorithms and hinder the selection of the top-performing ones. Although none of the algorithms included in this study were trained on ClinVar data, we found substantial overlap between our initial datasets and the training datasets of the following prediction algorithms: PMut, META-SNP, PredictSNP, PhD-SNP, SNPs&GO, PP-2-HumDiv, and PP-2-HumVar. Therefore, in order to investigate the potential impact of type 1 circularity on the performance of the abovementioned algorithms, we compared their performance on a dataset that included variants used to train these algorithms (CircD) and on a dataset that did not contain overlapping variants (EP) (Supplementary Table S7). It is pertinent to note that the prediction algorithms SIFT, PROVEAN, and PANTHER-PSEP were excluded from this analysis for the following reasons: SIFT was trained on lacI, lysosyme, and HIV protease amino acid substitutions, and so it does not contain overlapping variants with our benchmark datasets, while PROVEAN and PANTHER-PSEP lack training datasets.

**Figure 4:**
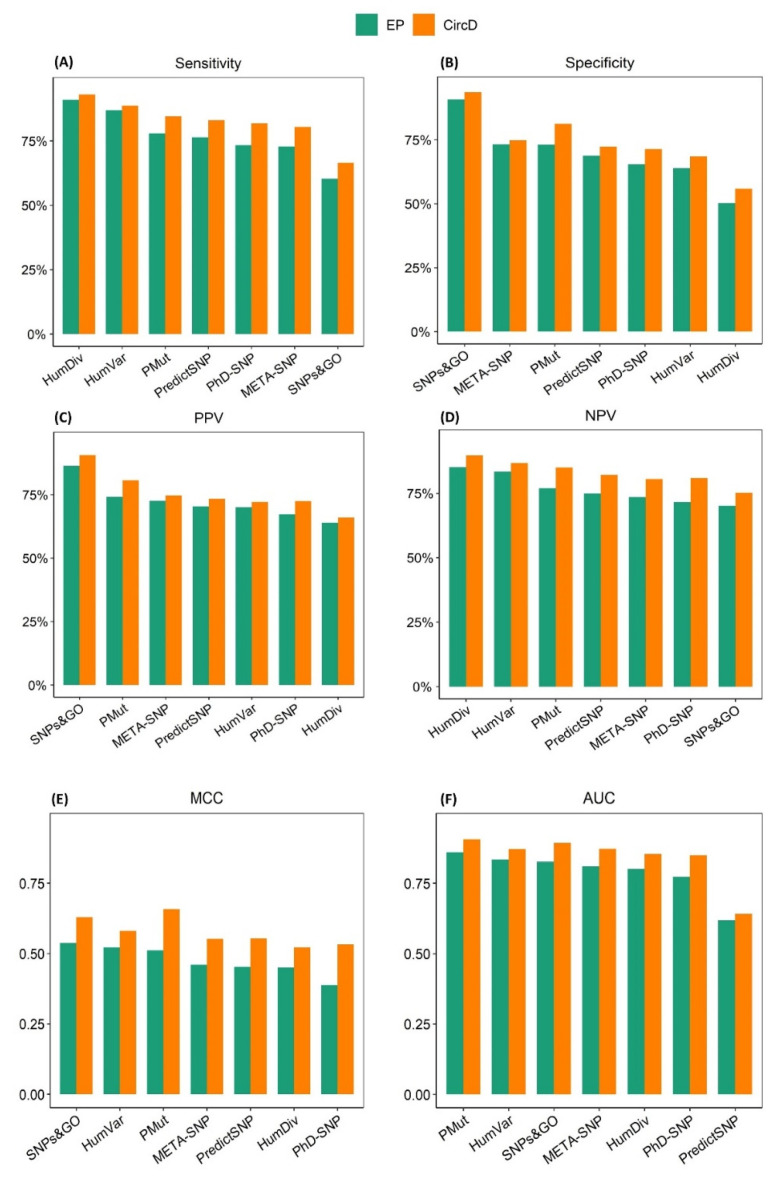
Comparison of the performance metrics of the prediction algorithms in the EP and CircD datasets. The CircD dataset was composed of 1053 missense variants, including those present in the training dataset of seven prediction algorithms. This dataset contained 505 pathogenic and 548 benign variants from 21 clinically relevant genes assigned to an expert panel review status in ClinVar. (**A**) Sensitivity; (**B**) Specificity; (**C**) PPV; (**D**) NPV; (**E**) MCC; (**F**): AUC. The prediction algorithms were ordered from top-performing to poor-performing based on their performance on the BRCA1 dataset. PPV, positive predictive value; NPV, negative predictive value; MCC, Matthews correlation coefficient; AUC, Area under curve.

As shown in Figure 4, the prediction algorithms performed better on the CircD dataset across all of the evaluated metrics. Although PMut, SNPs&GO, and PP-HumVar were confirmed to be the top-performing prediction algorithms based on their MCC and AUC values, their ranking order changed between the datasets. As shown in Figure 3E, PMut showed one of the highest increases in performance, advancing from third in the EP dataset to first in the CircD dataset, superseding SNPs&GO and HumVar. In addition, the ranking of the best performing prediction algorithms based on the AUCs changed substantially, with SNPs&GO and META-SNP ranking second and third, respectively, as compared to their respective third and fourth positions in EP (Supplementary Figure S4). Contrary to this, PP-2-HumVar fell from second to fourth in the CircD dataset despite showing an increase in its AUC value (Figure 4F). Overall, the median MCC and AUC values increased from 0.46 and 0.81, respectively, to 0.55 and 0.87 (Supplementary Figure S4). No major changes were observed in the order of the predictors in the other metrics (Table 2). Therefore, benchmarking datasets for the evaluation of the prediction algorithms must be carefully prepared in order to eliminate confusion when selecting the most accurate algorithms.

## 3. Discussion

In this study, we have systematically evaluated the predictive performance of ten commonly used prediction algorithms on four independent datasets compiled from the public database ClinVar [26], the Expert Panel (EP), and circularity (CircD) datasets composed of missense variants from 21 clinically relevant genes assigned to three-star status and the BRCA1 and BRCA2 datasets composed of a combination of BRCA1 and BRCA2 variants assigned to two- and three-star status in ClinVar.

According to our results, three machine learning-based prediction algorithms, HumVar, SNPs&GO, and PMut, displayed an overall above-average performance in the EP dataset as compared to the other algorithms. In contrast, PMut, SNPs&GO, and PANTHER-PSEP outperformed the other prediction algorithms in the BRCA1 dataset, whereas PMut was the best performing algorithm in the BRCA2 dataset.

As opposed to previous reports [20], the meta-predictors META-SNP and PredictSNP demonstrated an overall moderate performance across all datasets and did not show superior performance to the individual predictors.

Consistent with previous studies [11,30,32,34,40], we found that most of the evaluated prediction algorithms display higher sensitivities than specificities, suggesting that under current thresholds these algorithms tend to overcall variants as deleterious. This trend was particularly evident in SIFT, one of the most routinely used tools in clinical settings. As consistently shown across three benchmarking datasets, SIFT displayed excellent sensitivities (EP: 92.46%, BRCA1: 96.61%, BRCA2: 93.10%), outperforming all of the prediction algorithms, albeit with unacceptably low specificities (EP: 51.22%, BRCA1: 39.13%, BRCA2: 53.33%). In a clinical scenario, such low specificity values may result in more invasive treatments being recommended to patients carrying these false-positive variants [41]. The opposite was observed for SNPs&GO, which demonstrated the highest specificities in all datasets (EP: 90.73%, BRCA1: 92.31%, BRCA2: 98.10%) but at the cost of lower sensitivities (EP: 60.30%, BRCA1: 81.36%, BRCA2: 55.17%).

Furthermore, we found that across all datasets the NPVs were higher than the PPVs, notably in the BRCA1 and BRCA2 datasets, where 5 of 10 and 6 of 10 predictors, respectively, displayed NPV values >90%. This can be explained by the low prevalence of pathogenic variants in the BRCA1 and BRCA2 datasets, which were 39% and 22%, respectively. However, given the small number of pathogenic variants identified in the *BRCA* genes, the NPV values encountered in clinical settings are close to those reported in our study.

Additionally, our results show that the performance of some of the evaluated prediction algorithms varies from gene to gene. In our study, this observation was particularly evident in the function prediction algorithm PROVEAN, which falsely predicted the majority of the deleterious variants in the *BRCA*1 and *BRCA*2 genes as “neutral”. In contrast, PROVEAN could accurately predict deleterious variants for other tumor suppressors, such as *MSH*2, (22 of 23 deleterious mutations correctly predicted) as shown in the EP dataset, where PROVEAN displayed a sensitivity of 74.37%.

A possible explanation for the poor performance of PROVEAN in the *BRCA* genes could be that the homologs of the *BRCA*1 and *BRCA*2 genes are not highly conserved and have a low degree of similarity even among closely related species [42]. As an example, the human and mouse homologs of *BRCA*1 share only 56% of identity between each other, as compared to the 92% shared between the mouse *MSH*2 and its human homolog [42]. The low sequence similarity shared between the *BRCA* homologs may confound the core steps of the PROVEAN algorithm. This algorithm initially performs a BLAST [43,44] search to collect homologous sequences to the query; the retrieved sequences are then clustered according to a cut-off value of 75% sequence similarity within each cluster, and only 30 clusters with the highest similarity to the query are utilized to calculate the alignment scores to the query and mutation sequence as well as the PROVEAN scores [13]. Additionally, alignment accuracy can be drastically reduced in poorly conserved areas of the protein as well [41]. As a result, the low similarity that exists among the *BRCA* homologs could negatively affect the quality of the generated clusters and, consequently, the alignment and PROVEAN scores. Another possible explanation could be that the variant amino acid residue, instead of the reference amino acid, is found to be similar to the aligned amino acid in the homologous sequence, resulting in high delta scores [13].

A similar outcome from a different set of predictors was also reported by Marttoleto and colleagues [45], who found that some of the evaluated prediction algorithms showed a better performance for variants occurring in tumor suppressor genes, whereas others performed better for variants occurring in oncogenes. Our findings are also substantiated by a recent study conducted by Cubuk and colleagues, who compared 44 computational tools, including PROVEAN, on a unique dataset of missense variants from five tumor suppressor genes (*BRCA*1, *BRCA*2, *MSH*2, *PTEN*, and *TP*53) which had been clinically validated through high-throughput functional assays [40]. Based on the reported predictions, we verified that PROVEAN could correctly predict 4 out of 370 deleterious *BRCA*1 variants and 12 out of the 64 deleterious *BRCA*2 variants. By contrast, it correctly identified 352 of the 372 deleterious variants reported in MSH2.

Furthermore, our data undermine the current concordance-based approach for variant interpretation recommended by the ACMG/AMP guidelines (for details, see introduction) [17]. As we demonstrate here, the utilization of PROVEAN for variant interpretation in the *BRCA* genes could potentially disrupt the congruence of other prediction algorithms on the pathogenicity of a given variant, ultimately rendering the computational evidence inconclusive. Moreover, as previously reported, this discordance driven by the poor performance of some predictors can result in a higher VUS burden as well [19].

Another objective of this study was to investigate the potential impact of type 1 circularity on the performance of seven machine learning-based prediction algorithms, namely, PredictSNP, META-SNP, SNPs&GO, PMut, PP-2-HumDiv, PP-2-HumVar, and PhD-SNP. Based on our results, we found a notable increase in the performance of all of the prediction algorithms, leading to a shift in the order of the predictors as compared to the circularity-free dataset (EP). In agreement with previous studies [31], we demonstrated that, if not accounted for, type 1 circularity can lead to an unrealistic view of the performance of these seven predictors and can possibly confound tool selection.

Although ClinVar data have been used in several studies to benchmark the performances of various computational tools [19,20,30,40], our study differs in several aspects. First, our datasets were restricted, either solely to missense variants examined by an expert panel (assigned to a three-star review status) or to a combination of expert panel-reviewed variants and variants with available assertion criteria without conflict in interpretation (assigned to two-star review status). One-star variants were excluded due to the lower level of evidence associated with them as compared to the two- and three-star variants. For variants with a one-star rating, assertion criteria are provided by a single submitter or by multiple submitters but with conflicting interpretations [26]. Second, we investigated the possible impact of type 1 circularity on the performance of the following predictors: SNPs&GO, PredictSNP, META-SNP, PMut, and PhD-SNP, which—to the best of our knowledge—has not been previously reported. Third, our study included the updated version of PMut, which underwent a major update in 2017. In addition, we included PANTHER-PSEP, a relatively recent prediction algorithm developed in 2016 and not widely included in previous benchmarking studies.

According to our findings, prior to selecting a prediction algorithm, a thorough investigation of the literature and a critical examination of the reported evaluation metrics of the various predictors should be undertaken. Consulting a bioinformatician could also be beneficial, as prediction algorithms also go through major updates, as in the case of PMut [12]. Furthermore, given the increasing number of missense variants identified from patient samples and submitted to public databases such as ClinVar, we recommend that the developers of such algorithms incorporate such variants into the training data, thus diversifying the origin of the training datasets.

## 4. Materials and Methods

### 4.1. Variant Acquisition and Dataset Generation

**Figure 5:**
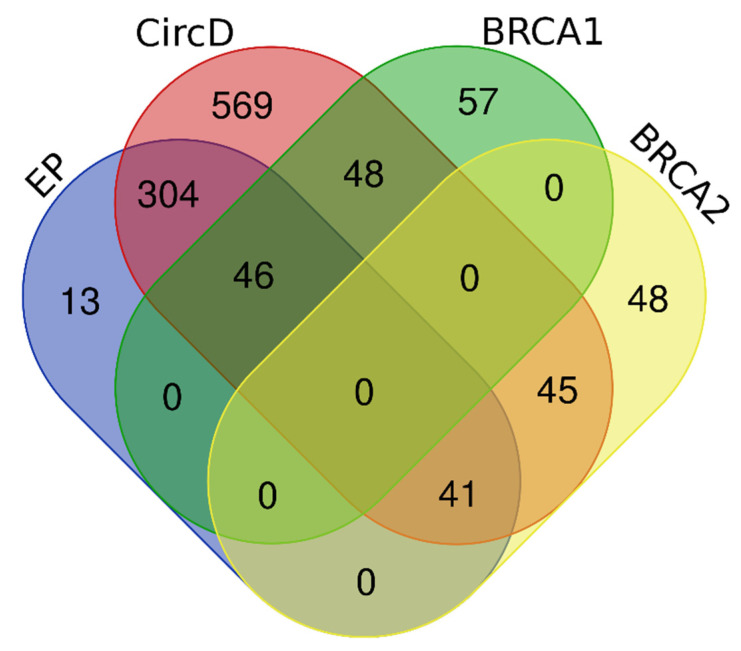
Wayne diagram representing the relationship between the EP, CircD, BRCA1, and BRCA2 datasets. The datasets were retrieved from the ClinVar database based on a stringent set of filtering criteria. The EP dataset and CircD datasets consisted of 404 and 1053 missense variants from 21 clinically relevant genes, respectively, whereas the BRCA1 and BRCA2 datasets were composed of 151 and 134 missense variants, respectively.

To evaluate the performance of the prediction algorithms, we compiled four independent datasets—their relationship is demonstrated in Figure 5—using missense variants with known clinical significance from the publicly available database ClinVar. A total of 1126 (534 pathogenic and 592 benign) variants from 21 genes were retrieved from the ClinVar database (last accessed: December 2021 to March 2022) based on the following criteria: (I) clinical significance: pathogenic/likely pathogenic and benign/likely benign; (II) molecular consequence: missense; and (III) review status: reviewed by an expert panel. The obtained variants were then filtered to remove duplicates and erroneously included non-missense variants. This filtering step resulted in 1053 missense variants (505 pathogenic and 548 benign), which were then screened against the training datasets of the following prediction algorithms: PredictSNP, PhD-SNP, META-SNP, HumDiv, HumVar, SNPs&GO, and PMut. All of the pathogenic and benign variants present in the training datasets of the abovementioned algorithms were removed to avoid inherent bias in their overall performance introduced by type 1 circularity [31]. To ensure that all predictors were evaluated on the same set of missense variants, those present in the training dataset of one algorithm but not the others were also excluded. Moreover, a portion of the benign variants in the *BRCA*1 and *BRCA*2 genes were randomly removed in order to reduce the imbalance between the pathogenic and benign variants. These steps resulted in 404 missense variants, 199 pathogenic and 205 benign variants, which were then utilized to form the expert panel dataset.

Additionally, due to the high degree of overlap between our initial dataset of 1053 filtered variants and the training datasets of the prediction algorithms, we generated an additional dataset, the circularity dataset. The circularity dataset was composed of the 1053 filtered missense variants and was utilized to analyze the potential effect of type 1 circularity on the performance of the prediction algorithms.

Furthermore, two additional benchmarking datasets were generated to compare the performance of the prediction algorithms in predicting missense variants in the *BRCA*1 and *BRCA*2 genes. The BRCA1- and BRCA2-specific datasets were created by collecting pathogenic or likely pathogenic and benign or likely benign missense variants for which assertion criteria by multiple submitters were available without conflicts of interpretation (assigned to a two-star status in ClinVar) and expert panel-reviewed variants (assigned to a three-star status in ClinVar). Initially, we obtained a total of 254 *BRCA*1 and 199 *BRCA*2 variants from the ClinVar database which were then processed and screened against the training datasets of the computational tools as described previously. For downstream analysis, we retained 151 *BRCA*1 (pathogenic: 59, benign: 92) and 134 *BRCA*2 variants (pathogenic: 29, benign: 105).

### 4.2. Dataset Composition

The datasets contained missense variants derived from a set of 21 clinically relevant genes, 14 of which were extracted from the PanCancer 405 gene panel provided by the diagnostics company Delta Bio 2000 Ltd., Szeged, Hungary. This panel—consisting of 405 genes—was screened against ClinVar, and only those genes that contained both pathogenic and benign expert panel-reviewed variants were included in the EP and CircD benchmarking datasets. Genes containing either only pathogenic or benign variants were filtered out in order to avoid potential bias introduced by type 2 circularity [31].

### 4.3. Prediction Algorithm Selection

Ten prediction algorithms were selected based on a stringent set of criteria: (I) requiring only amino acid changes as input; (II) availability of the training dataset(s); and (III) a minimum of 15 citations in peer-reviewed journals in the two-year period between 2020 and 2022 (excluding benchmarking studies). The last criterion aims to ensure the relevance of these computational tools in scientific research. The selected prediction algorithms include the eight individual predictors PANTHER-PSEP, PROVEAN, SIFT, Polyphen2 (HumDiv, HumVar), PMut, PhD-SNP, SNPs&GO, and two consensus predictors, META-SNP and PredictSNP.

META-SNP combines the output of four well-established predictors, namely, SIFT, PANTHER, SNAP, and PhD-SNP, whereas PredictSNP combines the input of six prediction algorithms: MAPP, PolyPhen-1, PolyPhen-2, PhD-SNP, SIFT, and SNAP. The main characteristics of these in silico tools are listed in Supplementary Figure S5, and a detailed description can be found in Supplementary Text File S1.

### 4.4. Benchmarking

The performance of ten in silico tools, namely, PANTHER-PSEP, PROVEAN, SIFT, HumDiv (Polyphen2), HumVar (Polyphen2), PMut, PhD-SNPs, SNPs&GOs, Meta-SNP, and PredictSNP, was evaluated for each dataset.

All of the computational tools, apart from SIFT and PhD-SNP, were accessed via their respective web interfaces and run using default parameters (Supplementary Text File S2). As input, all tools required either the amino acid sequence in FASTA format or the UniProt ID of the protein and the amino acid change. Predictions for PROVEAN, HumDiv, HumVar, and PMut were obtained through batch submissions, while SIFT and PhD-SNP prediction scores were retrieved from PROVEAN (Protein Batch Mode, Human) and SNPs&GO (All Methods), respectively.

We retrieved and utilized only canonical protein sequences from the UniProtKB database [46]. In the case of the *MECP*2 gene, missense variants in ClinVar were reported according to their position in the *MECP*2 Isoform B sequence. The “MVAGMLGLR” string of amino acids in the *MECP*2 canonical sequence (UniProt ID: P51608) differs from the “MAAAAAAAPSGGGGGGEEER” string that is present in the *MECP*2 Isoform B (Uniprot ID: P51608-2). In order to utilize the canonical sequence of *MECP*2, we found the corresponding amino acid positions, which were then utilized as input for the computational methods.

### 4.5. Variant Classification

Predictions for each dataset were generated utilizing author-recommended thresholds as indicated in their respective publications. META-SNP, PhD-SNP, PMut, and SNPs&GO classify variants into either “Disease” (score > 0.5) or “Neutral” (score ≤ 0.5) categories, whereas SIFT classifies variants with scores ≤ 0.05 as “Damaging” and those with scores above 0.05 as “Tolerated”.

PROVEAN applies a threshold of −2.5, where variants scoring ≤−2.5 are classified as “Deleterious” and those scoring >−2.5 as “Neutral”. By contrast, Polyphen2 (HumDiv and HumVar) classifies the variants into three different categories: probably damaging (0.85 to 1.0), possibly damaging (0.15 to 1.0), and benign (0.0 to 0.15). Similarly, PANTHER-PSEP classifies variants into probably damaging (preservation time >450 million years), possibly damaging (preservation time ranges between 200 and 450 million years), and probably benign (preservation time is less than 200 million years) based on a position-specific evolutionary preservation (PSEP) score. In addition to the PSEP score, PANTHER also outputs a probability score (pdel), which indicates the probability of the mutation affecting protein function. To facilitate the downstream analysis, the outputs from HumDiv, HumVar, and PANTHER were dichotomized by considering both probably damaging and possibly damaging variants as damaging. PredictSNP, on the other hand, considers variants that score within the interval [−1, 0] as “Neutral” and those within [0, +1] as “Deleterious”.

### 4.6. Performance Evaluation Metrics

Confusion matrices consisting of true positive (TP), false negative (FN), true negative (TN), and false positive (FP) values were created for each dataset. Variants that were correctly predicted as deleterious or damaging by the tools were classified as true positives, while those incorrectly predicted as benign or neutral were classified as false negatives. Correctly predicted benign or neutral variants were classified as true negatives, whereas those predicted as deleterious or damaging were classified as false positives.

Based on the generated confusion matrices, we evaluated the performance of the classifiers using seven metrics: accuracy, sensitivity, specificity, positive predictive value (PPV), negative predictive value (NPV), the area under the receiver operating characteristic (ROC) curve, and the Matthews correlation coefficient (MCC).

The prediction algorithms were categorized into two distinct categories: top-performing and poor-performing, based on their MCC and AUC values. The Matthews correlation coefficient was selected as one of the main classification criteria, since it weighs each class of the confusion matrix equally, and high values can only be generated if the algorithms are capable of correctly identifying cases in both classes (pathogenic and benign) [47]. Alternatively, the area under the curve (AUC) was chosen, as it determines how well a prediction algorithm is able to discriminate between benign and pathogenic variants, with values closer to 1 indicating a better ability of the prediction algorithms to differentiate neutral from the deleterious variants [48]. These metrics were calculated in R (version 4.2.0, R Foundation for Statistical Computing, Vienna, Austria) [49] using the cvms (cross-validation for model selection) package (version 1.3.3, Ludvig Renbo Olsen, Aarhus University, Aarhus, Denmark) [50]. The graphs were created using the R package ggplot2 (version 3.3.5, Hadley Wickham [51]. ROC curves and AUC values were generated using the Python library scikit-learn [52]. The utilized formulas were as follows:

(1) $$\mathrm{MCC}\;=\;\frac{\left(\mathrm{TP}\;\times \mathrm{TN}\right)\;-\;\left(\mathrm{FP}\;\times \;\mathrm{FN}\right)}{\sqrt{\left(\mathrm{TP}\;+\;\mathrm{FP}\right)\left(\mathrm{TP}\;+\;\mathrm{FN}\right)\left(\mathrm{TN}\;+\;\mathrm{FP}\right)\left(\mathrm{TN}\;+\;\mathrm{FN}\right)}}$$

(2) $$\mathrm{Accuracy}\;=\;\frac{\mathrm{TP}\;+\;\mathrm{TN}}{\mathrm{TP}\;+\;\mathrm{FP}\;+\;\mathrm{TN}\;+\mathrm{FN}}$$

(3) $$\mathrm{Sensitivity}\;=\;\frac{\mathrm{TP}}{\mathrm{TP}\;+\;\mathrm{FN}}$$

(4) $$\mathrm{Specificity}\;=\;\frac{\mathrm{TN}}{\mathrm{TN}\;+\;\mathrm{FP}}$$

(5) $$\mathrm{PPV}\;=\;\frac{\mathrm{TP}}{\mathrm{TP}\;+\;\mathrm{FP}}$$

(6) $$\mathrm{NPV}\;=\;\frac{\mathrm{TN}}{\mathrm{FN}\;+\;\mathrm{TN}}$$

## 5. Conclusions

To conclude, our results emphasize the importance of systematic benchmarking of computational tools on novel datasets composed of variants with a high degree of confidence. The performance of the prediction algorithms varied considerably across the benchmarking datasets. Accordingly, for the EP dataset, HumVar, SNPs&GO, and PMut displayed above-average MCC and AUC values, outperforming all of the other prediction algorithms analyzed in this study. In contrast, PANTHER-PSEP, PMut, and SNPs&GO were determined to be the best performing prediction algorithms in the BRCA1 datasets, whereas PMut was determined to be the best performing prediction algorithm for the BRCA2 dataset. Due to their ability to accurately predict both pathogenic and benign missense variants in the *BRCA*1 and *BRCA*2 genes, we strongly recommend these algorithms for the classification of missense variants in these two genes.

Additionally, we recommend avoiding the use of PROVEAN for prioritizing variants in the *BRCA*1 and *BRCA*2 genes, since it would hinder the congruence between the utilized prediction algorithms, rendering the computational evidence futile. Furthermore, using a specific dataset (CircD), we demonstrated that seven of the evaluated tools were notably confounded by type 1 circularity, which, if not addressed prior to benchmarking the prediction algorithms, can lead to an artificial increase in their performance and, consequently, to selection of the wrong algorithms.

## Figures and Tables

**Table 1:** Performance evaluation metrics on the (A) BRCA1 and (B) BRCA2 Datasets.

Software	ACC (%)	SEN (%)	SPE (%)	PPV (%)	NPV (%)	MCC	AUC
**(A)** **BRCA1**							
**PMut**	85.43	88.14	83.7	77.61	91.67	0.705	0.932
**PROVEAN**	42.38	0.00	69.57	0.00	52.03	−0.382	0.507
**SIFT**	61.59	96.61	39.13	50.44	94.74	0.402	0.910
**SNPs&GO**	88.08	81.36	92.39	87.27	88.54	0.748	0.925
**PhD-SNP**	68.87	93.22	53.26	56.12	92.45	0.475	0.893
**PredictSNP**	72.85	93.22	59.78	59.78	93.22	0.530	0.773
**META-SNP**	74.17	91.53	63.04	61.36	92.06	0.540	0.873
**PAN-PSEP**	88.08	83.05	91.3	85.96	89.36	0.748	0.895
**HumDiv**	57.62	72.88	47.83	47.25	73.33	0.206	0.660
**HumVar**	60.26	52.54	65.22	49.21	68.18	0.176	0.650
**(B)** **BRCA2**							
**PMut**	93.28	75.86	98.10	91.67	93.64	0.794	0.898
**PROVEAN**	63.43	13.79	77.14	14.29	76.42	−0.092	0.544
**SIFT**	61.94	93.10	53.33	35.53	96.55	0.386	0.859
**SNPs&GO**	88.81	55.17	98.10	88.89	88.79	0.643	0.801
**PhD-SNP**	78.36	65.52	81.90	50.00	89.58	0.433	0.795
**PredictSNP**	76.12	82.76	74.29	47.06	93.98	0.484	0.678
**META-SNP**	79.85	65.52	83.81	52.78	89.80	0.458	0.805
**PAN-PSEP**	73.88	86.21	70.48	44.64	94.87	0.473	0.850
**HumDiv**	69.40	89.66	63.81	40.63	95.71	0.441	0.874
**HumVar**	79.10	82.76	78.10	51.06	94.25	0.525	0.902

ACC: accuracy, SEN: sensitivity, SPE: specificity, PPV: positive predictive value, NPV: negative predictive value, MCC: Matthews correlation coefficient, AUC: area under the PAN-PSEP, PANTHER-PSEP.

**Table 2:** Performance evaluation of the prediction algorithms in the CircD dataset.

Software	ACC (%)	SEN (%)	SPE (%)	PPV (%)	NPV (%)	MCC	AUC	M/V
**PMut**	82.84	84.54	81.26	80.65	85.05	0.657	0.905	16
**SNPs&GO**	80.63	66.53	93.61	90.57	75.22	0.629	0.892	0
**PhD-SNP**	76.35	81.78	71.35	72.46	80.95	0.533	0.848	0
**PredictSNP**	77.40	82.97	72.26	73.38	82.16	0.554	0.641	0
**META-SNP**	77.49	80.40	74.82	74.63	80.55	0.552	0.871	0
**HumDiv**	73.69	93.04	55.86	66.01	89.71	0.522	0.853	0
**HumVar**	78.17	88.67	68.50	72.17	86.77	0.580	0.870	0

ACC: accuracy, SEN: sensitivity, SPE: specificity, PPV: positive predictive value, NPV: negative predictive value, MCC: Matthews correlation coefficient, AUC: Area under the receiver operating characteristic curve (ROC), M/V: missing values.

## Data Availability

The data presented in this study are available as a supplement. The gene list of the PanCancer 405 gene panel is available from the corresponding author upon request. The data are not publicly available due to copyright.

## Supplementary Materials

The following supporting information can be downloaded at: https://www.mdpi.com/article/10.3390/ijms23147946/s1.
