# Supplementary material for: A Comprehensive Evaluation of the Performance of Prediction Algorithms on Clinically Relevant Missense Variants
Source: Int J Mol Sci. 2022 Jul 19;23(14):7946. doi: 10.3390/ijms23147946 (PMC9322961; doi:10.3390/ijms23147946)
Supplement: Supplementary file 1 [file ijms-23-07946-s001.zip › Supplementary Figure S5.pdf]

Software	Predictive Model	Features
PROVEAN	Delta Alignment Score	Evolutionary Conservation
PANTHER	Preservation score (PSEP Score)	Evolutionary Preservation
META-SNP	Random Forest	Combines the tools: SNAP, PhD-SNP, Panther, and SIFT
SNPs&GO	Support Vector Machines	Sequence-homology and GO terms
PredictSNP	Random Forest	Combines the tools: MAPP, PhD-SNP, Polyphen1, Polyphen2, SIFT, SNAP
HumDiv	Naive Bayes	Sequence homology, and structural features
HumVar	Naive Bayes	Sequence homology and structural features
SIFT	SIFT Alignment Score	Sequence homology and physical properties of amino acids
PMut	Random Forest	Amino acid conservation, protein interactions, physical properties
PhD-SNP	Support Vector Machines	Evolutionary Conservation
