# Supplementary material for: A Comprehensive Evaluation of the Performance of Prediction Algorithms on Clinically Relevant Missense Variants
Source: Int J Mol Sci. 2022 Jul 19;23(14):7946. doi: 10.3390/ijms23147946 (PMC9322961; doi:10.3390/ijms23147946)
Supplement: Supplementary file 1 [file ijms-23-07946-s001.zip › Supplementary Text File S1.pdf]

File S1

A description of the ten benchmarked prediction algorithms.

1. **PMut (PMut2017)**

PMut is a random forest-based classifier that was trained with 12 selected features on the SwissVar (October 2016) dataset containing 27,203 pathogenic mutations and 38,078 benign mutations originating from 12,141 proteins. PMut calculates various features, including sequence conservation; protein interactome; differences in physical properties of the wild-type and the variant amino acid, such as hydrophobicity; position in the protein sequence; Kyte–Doolittle hydropathy index; and the relative and absolute difference in volume in order to determine the impact of an amino acid substitution on protein function [12]. A score ranging from [0, 1] is assigned to each predicted variant and is classified as either "Neutral" (from 0 to 0.5) or "Disease" (from 0.5 to 1).

2. **PROVEAN**

PROVEAN (Protein Variation Effect Analyzer) is a multi-functional computational method that, in addition to amino acid substitutions, can also be utilized to predict the effect of deletions and in-frame insertions on protein function. PROVEAN is based on the assumption that the occurrence of variations that reduce the similarity between the query protein and its homolog are more likely to have a detrimental effect on the protein's function. Delta, an alignment-based score, is computed and used as a metric for measuring the differences in the similarity between the target sequence and its homologous sequences before and after an amino acid change occurs in the target protein [13]. PROVEAN uses a cut-off of -2.5 to classify variants, where variants scoring less than or equal to -2.5 are classified as "Deleterious" and those scoring above -2.5 as "Neutral".

3. **SIFT**

SIFT (Sorting Intolerant from Tolerant) determines whether the substitution of an amino acid will alter the function of a protein based on sequence homology and the physical properties of amino acids [9]. SIFT assumes that if an amino acid is conserved in a protein family, its substitution will have a detrimental effect on protein function. For instance, if an alignment contains hydrophobic amino acids, it is assumed that this position can only contain hydrophobic amino acids, and changes to other hydrophobic amino acids are predicted to be tolerated, but changes to amino acids with different physical properties, such as polar amino acids, will have a detrimental effect on protein function. Using the target protein sequence as a query, the SIFT algorithm carries out a database search to collect homologous sequences [9]. Of the collected sequences, only those with an adequate sequence diversity are kept and aligned. A SIFT score is calculated which ranges from 0 to 1 and indicates the normalized probability of finding the new amino acid at the specified position. Variants with scores between 0 and 0.05 are predicted as "Damaging", while those with scores between 0.05 and 1.0 are predicted as "Tolerated".

4. SNPs&GO

SNPs&GO is a support vector machine (SVM)-based predictor that combines sequence information with functional information from GO terms (Molecular Function, Biological Process, Cellular Components) to predict deleterious amino acid variants [36]. The SNPs&GO algorithm calculates a 51-element feature vector for each amino acid variation which comprises features such as the mutation, sequence profile, sequence environment, number of GO terms, log odd scores, and four features from the PANTHER output. In cases where the PANTHER algorithm does not return an output, an arbitrary input vector is used. This vector assigns a probability of 0.5 for deleterious variants and 0 for the other three PANTHER features. The model was trained on 38,460 disease-associated and neutral SNVs derived from 9,067 proteins in the SwissVar dataset (October 2009) and uses a cut-off value of 0.5 to classify variants as "Disease" (>0.5) or "Neutral" (≤0.5).

5. PhD-SNP

PhD-SNP utilizes three different methods for analyzing whether a missense variant is disease-associated or neutral: (i) the baseline predictor (ProbMeth); (ii) the sequence-profile support vector machine (SVM) method (SVM-Profile); and (iii) the sequence-profile support vector machine (SVM-Sequence) [35]. Initially, the SVM-Sequence method classifies variants into disease-associated and neutral categories based on a cut-off value of 0.5, followed by the SVM-Profile method, which classifies variants into disease and neutral using the two-element vector from the sequence profile, whereas the hybrid method (HybridMeth) utilizes a decision tree model with SVM-Sequence coupled to the SVM-Profile. PhD-SNP was trained on two datasets: the SVP-Sequence method was trained on the HumVar dataset, and the SVM-Profile method was trained on HumVarProf. PhD-SNP applies a cut-off value of 0.5, where values equal to or above 0.5 are classified as "Disease" and those below 0.5 as "Neutral".

6. PredictSNP

PredictSNP is a consensus predictor developed using a random forest model that incorporates the output of six independent tools, MAPP, PolyPhen-1, PolyPhen-2, PhD-SNP, SIFT, and SNAP, to distinguish between disease-associated and neutral missense variants [23]. PredictSNP computes a confidence score for every variant that ranges from -1 to 1 and then converts it into observed accuracy values. Variants scoring between -1 and 0 are considered "Neutral" and those scoring between 0 and 1 as "Deleterious".

7. META-SNP

META-SNP is a meta-predictor that combines the output of four well-established individual predictors, namely, PANTHER, SIFT, PhD-SNP, and SNAP, to distinguish between disease-associated and neutral variants. As an input, META-SNP takes a feature vector of eight elements composed of two groups of four elements each. The first group contains the raw output scores of PANTHER, PhD-SNP, SIFT, and SNAP, whereas the second group includes four elements extracted from the PhD-SNP protein sequence profile [15]. This random forest binary classifier was trained on a balanced dataset consisting of 35,766 disease-causing and neutral missense variations from 8,667 proteins (SwissVar dataset, released in October 2009) (Capriotti, Altman, et al., 2013). By applying a threshold score of 0.5, variants with scores equal to or above 0.5 are classified as "Disease" and those with scores below 0.5 as "Neutral".

8. PANTHER-PSEP

PANTHER-PSEP employs evolutionary preservation to predict the potential impact of nsSNVs on the biological function of a given protein. In PANTHER-PSEP, evolutionary preservation is determined by the length of a site that has been conserved in the target sequence by tracing it back to the lineage leading to the sequence of interest [37]. PANTHER-PSEP computes the time of preservation in millions of years (my), which is used to categorize the nsSNVs into probably damaging (> 450 my), possibly damaging (200 to 450 my), and probably benign (< 200 my) groups. Additionally to the PSEP score, the algorithm also calculates a pdel score, which is a measure of the likelihood that the mutation has a deleterious effect on protein function.

9. PolyPhen-2- HumDiv

Polyphen2 uses a Naïve Bayes classifier to predict the potential effects of an amino acid substitution on protein function. Polyphen2 combines a multitude of sequence and structural features, such as electrostatic interaction, ligand interaction, hydrophobic tendency, solvent accessibility, etc. [8]. Polyphen2 offers two Naïve Bayes probabilistic models: HumDiv and HumVar, both trained on two different datasets.

HumDiv is the default classifier trained on the HumDiv dataset and recommended for evaluating rare alleles located at loci that may be involved in complex phenotypes. This classifier was trained on the HumDiv dataset, which consists of damaging alleles found in the UniProtKB database that have known effects on molecular function, causing human Mendelian diseases.

10. PolyPhen-2- HumVar

The HumVar classifier was trained using all human disease-causing mutations from UniProtKB as well as common SNPs (MAF>1%) that were not treated as harmful mutations by UniProtKB. It is recommended for diagnosing Mendelian diseases, which require distinguishing mutations with drastic effects from all the remaining human variations, including abundant mildly deleterious alleles.

Each analyzed variant in Polyphen2 is then assigned a prediction outcome and a probabilistic score. The Polyphen2 score ranges from 0.0 to 1.0, with variants scoring from 0.0 to 0.15 classified as "benign" and those scoring from 0.15 to 1.0 as "possibly damaging". Polyphen2 describes a third class of variants that are predicted to be "probably damaging" and assigned scores between 0.85 and 1.0.
