# Supplementary material for: A Comprehensive Evaluation of the Performance of Prediction Algorithms on Clinically Relevant Missense Variants
Source: Int J Mol Sci. 2022 Jul 19;23(14):7946. doi: 10.3390/ijms23147946 (PMC9322961; doi:10.3390/ijms23147946)
Supplement: Supplementary file 1 [file ijms-23-07946-s001.zip › Supplementary Text File S2.pdf]

File S2

A detailed description of the running parameters and the input format utilized to generate the predictions for all of the datasets:

1. **PROVEAN**

PROVEAN was accessed via the web interface (http://provean.jcvi.org/index.php), version 1.1.3. The analysis was performed using PROTEIN BATCH Mode (Human), with default settings, which included the UniProt ID of the query protein and amino acid changes according to the following format:
<UniProt ID>, <position>, <reference amino acid>, <variant amino acid>

2. **SIFT**

SIFT predictions were obtained from the PROVEAN Protein Batch Mode, which generates them in addition to the PROVEAN predictions.

3. **SNPs&GO**

SNPs&GO was accessed through the web interface (https://snps.biofold.org/snps-and-go//snps-and-go.html) and run with default settings. The "All Methods" option, which returns the predictions for PhD-SNP and PANTHER was enabled. The default settings included the query protein sequence in Fasta format and the amino acid changes according to the following format:
<Protein Sequence>, <A1PosA2> where **A1** is the reference amino acid, **A2** is the variant amino acid, and **Pos** is the position of the reference amino acid in the protein sequence.

4. **PhD-SNP**

The PhD-SNP predictions were obtained using the "All Methods" mode on SNPs&GO. A batch query option was not available on the PhD-SNP's web interface.

5. **PMut**

The PMut predictions were generated via the web interface (http://mmb.irbbarcelona.org/PMut/) using the Protein Batch Mode with default settings. Default settings included: the UniProt ID of the target proteins and the amino acid change in the following input format:
<Uniprot ID>, <WT><Position><MT>

6. **PANTHER-PSEP**

PANTHER-PSEP (version 9.0) was accessed through the web interface (http://www.pantherdb.org/tools/csnpScore.do), and predictions were generated using default settings, with the "Select Single Organism" option set to "Human". As a default setting, the amino acid sequences of the target proteins and the amino acid changes were entered in the following format:
<amino acid sequence>, <A1PosA2> where **A1** is the reference amino acid, **A2** is the variant amino acid, and **Pos** is the position of the reference amino acid in the protein sequence.

7. META-SNP

META-SNP was accessed at (http://snps.biofold.org/meta-snp/), and the variants were analyzed using the default settings: amino acid sequence in Fasta format and the amino acid changes according to the following format:
<Amino acid sequence>, <A1PosA2> where **A1** is the reference amino acid, **A2** is the variant amino acid, and **Pos** is the position of the reference amino acid in the protein sequence.

8. PredictSNP

PredictSNP predictions were generated using the web interface (https://loschmidt.chemi.muni.cz/predictsnp1/) with default settings: amino acid sequence and amino acid change in the following input format:
<Amino acid sequence>, < A1PosA2 > where **A1** is the reference amino acid, **A2** is the variant amino acid, and **Pos** is the position of the reference amino acid in the protein sequence.

9. Polyphen-2-HumDiv

Polyphen-2-HumDiv was accessed via the web interface (http://genetics.bwh.harvard.edu/pph2/bgi.shtml), and predictions were generated using the Batch Query mode. According to the author's recommendations, the default settings were as follows:
Classifier model: HumDiv
Genome assembly: GRCh37/hg19
Transcripts: Canonical
Annotations: Missense

10. Polyphen-2-HumVar

Polyphen-2-HumVar predictions were generated through the web interface (http://genetics.bwh.harvard.edu/pph2/bgi.shtml), using the Query Batch mode with default settings, as follows:
Classifier model: HumVar
Genome assembly: GRCh37/hg19
Transcripts: Canonical
Annotations: Missense
As an input, Polyphen-2-HumDiv and Polyphen-2-HumVar required the UniProt ID of the target proteins and the amino acid changes according to the following format:
<UniProt ID>, <reference amino acid position>, <reference amino acid>, <variant amino acid>
