# Supplementary figures and images for: A Comprehensive Evaluation of the Performance of Prediction Algorithms on Clinically Relevant Missense Variants
Source: Int J Mol Sci. 2022 Jul 19;23(14):7946. doi: 10.3390/ijms23147946 (PMC9322961; doi:10.3390/ijms23147946)

ROC - EP

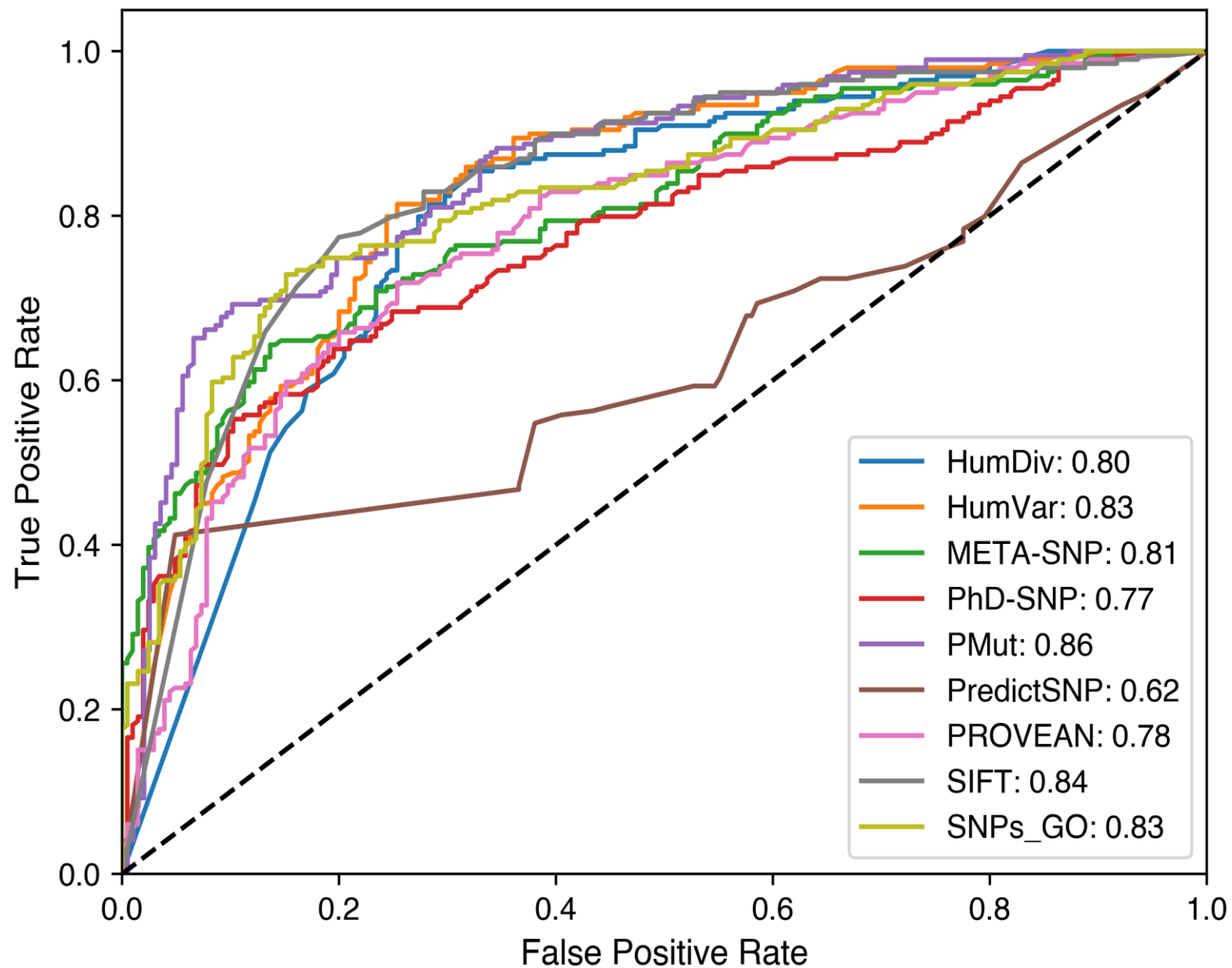

Supplement: Supplementary file 1 [file ijms-23-07946-s001.zip › Supplementary Figure S1.pdf]

ROC - BRCA1

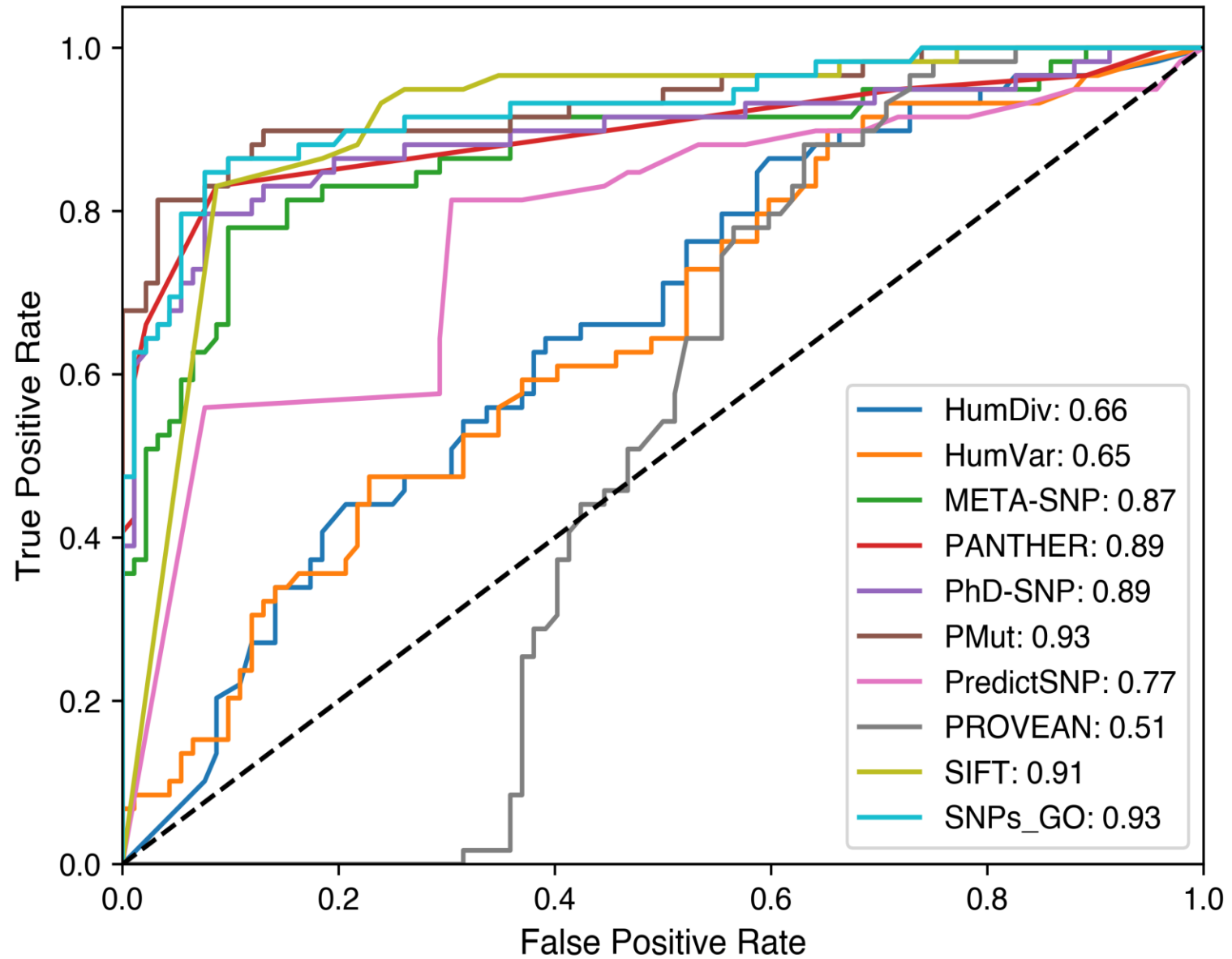

Supplement: Supplementary file 1 [file ijms-23-07946-s001.zip › Supplementary Figure S2.pdf]

ROC - BRCA2

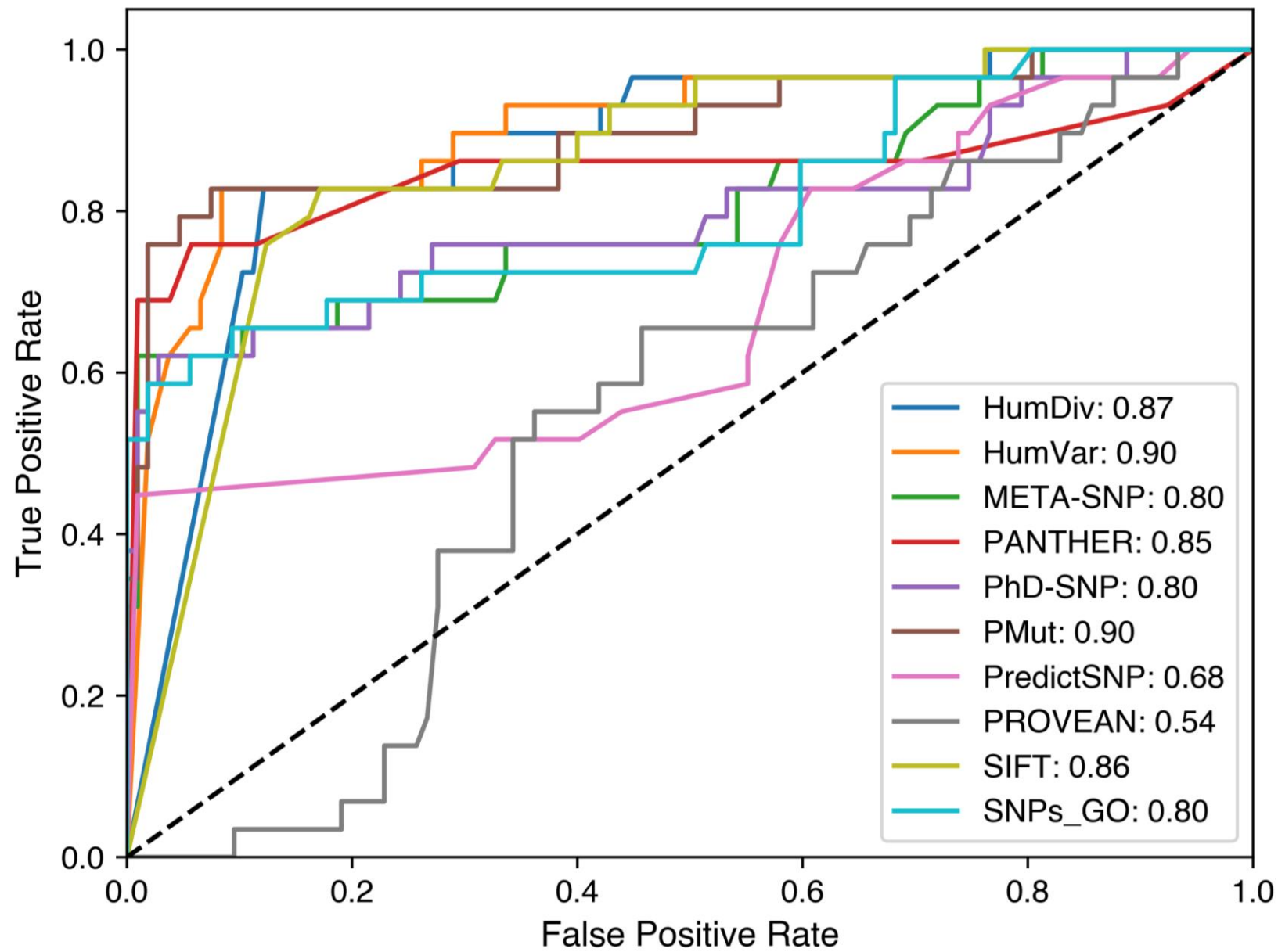

Supplement: Supplementary file 1 [file ijms-23-07946-s001.zip › Supplementary Figure S3.pdf]

ROC - CircD

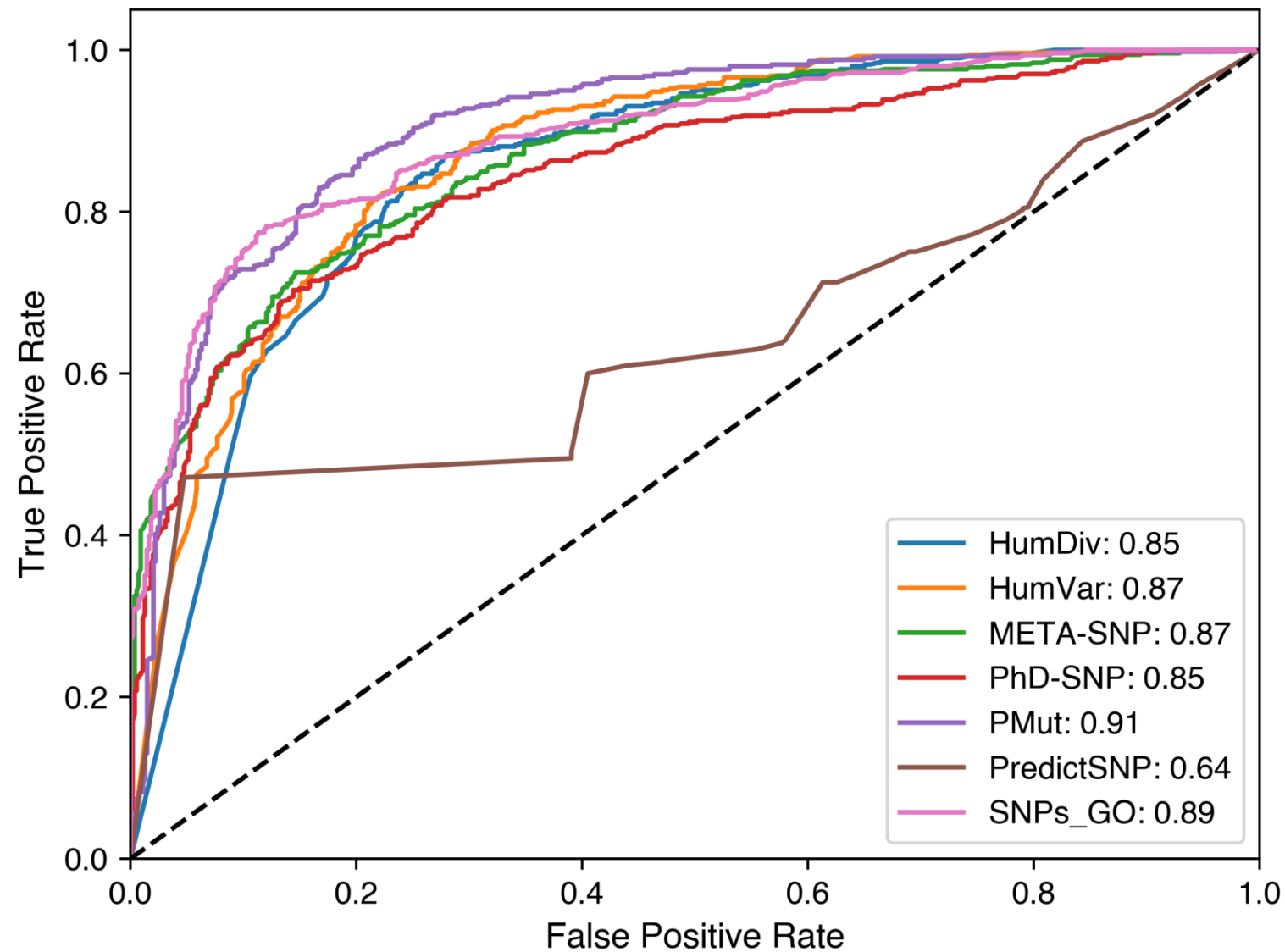

Supplement: Supplementary file 1 [file ijms-23-07946-s001.zip › Supplementary Figure S4.pdf]
